# Can emerging technologies be effective in improving alexithymia due to brain lesion? Lessons from a case report

**DOI:** 10.1097/MD.0000000000022313

**Published:** 2020-09-18

**Authors:** Rosaria De Luca, Francesca Sciarrone, Alfredo Manuli, Michele Torrisi, Bruno Porcari, Carmela Casella, Alessia Bramanti, Rocco Salvatore Calabrò

**Affiliations:** a Robotic and Behavioral Laboratory - IRCCS Centro Neurolesi “Bonino-Pulejo”; b Stroke Unit, University of Messina, Italy.

**Keywords:** alexithymia, case report, innovation technologies, ischemic stroke, virtual reality

## Abstract

**Introduction:**

About 66% of stroke survivors present with cognitive or physical consequences, which are often complicated by emotional instability. Alexithymia is defined as “a difficulty in identifying and describing feelings”, although there is no consensus on the exact diagnosis and treatment.

**Patient concerns:**

A 36-year-old right-handed man, affected by ischemic stroke (which occurred about 3 months before admission) involving the right hemisphere (ie, the fronto-parieto-temporal region) with left hemiparesis and behavioral abnormalities, came to our observation for intensive rehabilitation. He was treated unsuccessfully with a traditional and behavioral training.

**Diagnosis:**

Alexithymia due to ischemic stroke.

**Interventions:**

Then, a specific combined protocol using computerized emotional and virtual emotional training was applied in a semi-immersive virtual reality environment using the BTS-Nirvana device.

**Outcomes:**

At the end of this novel rehabilitation approach, the patient showed a significant improvement in emotional skills, cognitive performances, and coping strategies.

**Conclusions:**

Virtual reality, in addition to standard therapy, may be a valuable tool in improving emotional abnormalities due to brain lesions, such as alexithymia.

## 1 Introduction

The World Health Organization defines stroke as: “rapidly developing clinical signs of focal (or global) disturbance of cerebral function, with symptoms lasting 24 hours or longer or leading to death, with no apparent cause other than of.” Stroke is considered the second leading cause of death, and the third main cause of disability in both developed and developing countries.^[1,2]^ Such neurological disease can cause important organic, psychological and emotional disturbances, such as pseudobulbar affect (PBA) and emotional dysregulation.^[3]^ PBA includes pathological laughing and crying, emotional liability, emotional dysregulation, involuntary emotional expression disorder, and emotional incontinence. Among these disorders, alexithymia should not be confused with PBA or pathological laughing and crying. By definition, alexithymia is the “reduced ability to identify and refer to emotional states, as well as an impoverished fantasy life”.^[4]^

Nemiah and Sifneos initially used the term “no words for feelings” to identify the psychological construct of alexithymia, which is now considered as a cluster of cognitive traits, including difficulty in identifying and describing feelings to others, with reduced imaginative capacity.^[5]^ Despite alexithymia is not classified as a mental disorder in DSM-5, it is defined as a trans-diagnostic and dimensional personality construct, given that it is normally distributed among the general population, with a percentage of about 10%.^[6]^ Alexithymia is a common condition in patients with neurological diseases. Recent studies have focused on alexithymia in post stroke patients, showing that patients with higher levels of alexithymia presented co-morbidities with other psychological disorders including depression and anxiety, which altered their emotional status by adopting improper regulation strategies.^[7]^

To this end, it is possible to consider alexithymia as a risk factor for the development of post-stroke depression, as this predisposition may emerge after the traumatic organic events.^[8]^

Wang, et al. showed that alexithymic characteristics affect the psychological outcomes immediately after stroke, but not in the long term. Moreover, they found that post-traumatic stress disorder might develop in the presence of alexithymia traits.^[9]^ Spalletta et al demonstrated in a sample of 48 stroke patients that right brain damage contributes to the development of alexithymia (total Toronto Alexithymia Scale (TAS-20) score or TAS subscale score) more than left brain damage.^[10]^ Alexithymia was related to poorer outcomes, even if cognitive behavioral therapy (CBT) has proven effective for the treatment of the disorder.^[11]^ A randomized clinical trial has shown that computerized CBT may positively affect alexithymia patients’ outcomes, as computerized therapies reduce the need of applying interpersonal skills and interactions.^[12]^ In fact, growing data are demonstrating the effectiveness of computerized CBT, as compared to conventional CBT, supporting the idea that this tool might be used in several settings and different patient populations.^[11–15]^ The use of computerized training has been recently validated as a promising tool in the rehabilitation of brain injuries, including stroke, whereas growing evidence is demonstrating that the use of a semi-immersive virtual reality (VR) environment accelerates the rehabilitation process of compromised cognitive functions.^[16]^ Regarding the efficacy of new technologies, several studies show that the use of advanced tools using VR, both immersive (such as BTS- Nirvana -BTS-N) and non-immersive (using the robotic devices Lokomat or Armeo-P), improves the effects of the ongoing conventional neurorehabilitation, so to be considered complementary treatments in post - stroke physical, cognitive and psychological recovery.^[17–20]^ Moreover, VR treatments can increase patient's safety and self-efficacy, as well as coping strategies, eventually leading to an improvement in motivation and rehabilitation outcomes.

For this reason, in this case report, we attempted to evaluate the effects of an innovative combined rehabilitative program, using Computerized Emotional Training (CET) and Virtual Emotional Training (VET) with BTS-N in a post-stroke alexithymic patient.

## 2 Case description

A 36-year-old right-handed man, affected by ischemic stroke (which occurred about 3 months before admission) involving the right hemisphere (ie, the fronto-parieto-temporal region) with left hemiparesis, came to our observation for intensive rehabilitation. Psychomotor development was normal and he reported graduating from secondary school. In the last 10 years, he has been affected by dilative cardiomyopathy, diabetes mellitus and obesity. No previous mental illness was reported.

During the hospitalization at the Neurorehabilitation Unit of IRCCS Neurolesi (Messina, Sicily), patient's cognitive and behavioral symptoms, including depressive mood and apathy, were detected and treated by psychological counseling and serotoninergic antidepressants (ie, escitalopram 10 mg/daily without neither significant relief from depression nor adverse events). However, during the first 5 months of his hospitalization, after having undergone intensive conventional rehabilitation, there was no significant emotional and cognitive recovery at the end of this first rehabilitative cycle. Conventional cognitive and behavioral rehabilitation (a total of 30 sessions, 3 times a week, and each session lasting 45 minutes) was administered using only a traditional setting (face to face with the therapist) and a paper and pencil approach (Table 1).

**Table 1:**
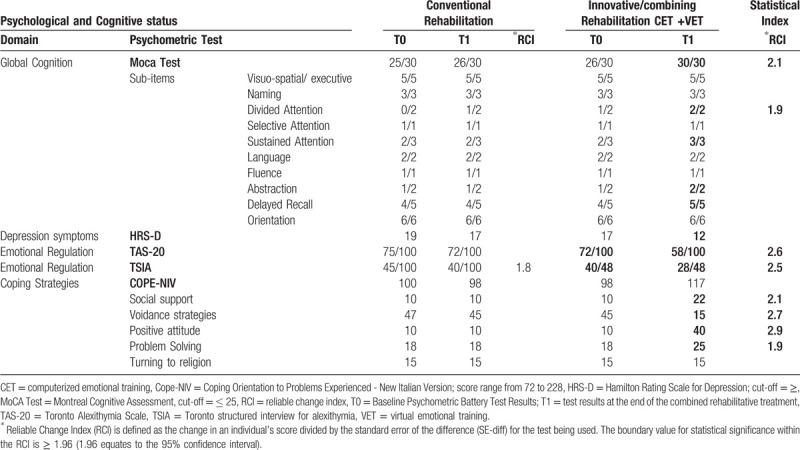
Comparison of behavioral and psychometric measures after the conventional and the innovative approach.

Moreover, the setting was adapted to the patient's needs, and nurses were trained to properly manage his symptomatology.

Motor recovery was instead significant, and the patient was able to walk with a cane and achieve autonomy in some daily living activities. For this reason, eight months after admission to hospital, we decided to stimulate the residual cognitive and emotional resources through a specific innovative approach, combining computer-based and virtual tools.

To evaluate the neuropsychological status, we used a specific psychometric battery investigating attention, memory, learning, executive functions, and mood. The psychometric battery included the Montreal Cognitive Assessment to assess global cognitive status, Hamilton Rating Scale for Depression to evaluate mood, 20-item TAS-20 and Toronto Structured Interview for Alexithymia (TSIA) to assess the severity of alexithymia.^[21–24]^

## 3 Written informed consent was obtained from the patient for publication of this case report and accompanying images

### 3.1 Outcome Measures and Interventions

TAS-20 is a self-report questionnaire composed of 20 items that participants endorse on a 5-point Likert-type scale, ranging from “strongly disagree” to “strongly agree.” The total score can range from 20 to 100. A score of 61 or more is taken to be diagnostic of alexithymia; 52 to 60 indicates “possible” alexithymia; 51 or less indicates the absence of alexithymia. In addition to the total score, 3 sub scores, which measure distinct factors: “difficulty identifying emotions”, “difficulty describing emotions”, and “externally oriented thinking” (EOT) can be calculated.

TSIA is a semi-structured interview that investigates the difficulty to identifying feelings, the difficulty to describing feelings, the externally oriented cognitive style of thinking (EOT); the fantasy and imaginative processes (IMP). The Coping Orientation to Problems Experienced - New Italian Version (COPE-NIV) was used to assess coping strategies. This version is a very valid and useful tool to measure coping styles in the Italian population. The COPE-NIV consists of 5 dimensions: social support, avoidance strategies, positive attitude, problem solving, and turning to religion.

The psychometric battery was administered at baseline (T0) and at the end of combined rehabilitative treatment (T1).

This innovative rehabilitation program included a combined training consisting of CET and VET with BTS-N. Each training was articulated in 2 sessions/wk (ie 2 CET and 2 VET per week) for 8 weeks and a total of 32 sessions. Each session lasted about 45 minutes, and was characterized by:

(1) tasks of discrimination of the emotional prosody from patted events (emotional discrimination);
(2) tasks of identifying the emotional significance of prosodic indices compared to verbal labels or facial expressions (*emotional identification*);
(3) expression of a judgment on prosodic stimuli by dimensions such as valence or intensity (*emotional evaluation*).

During CET, we presented male and woman faces with neutral, happy, angry, fearful, sad, disgusted and surprised expressions (*emotional identification*). Then, the patient was invited to recognize the emotions and to talk about the emotional items presented (*emotional discrimination*). Next, we asked to describe the emotions mostly felt by patient. Finally, we presented natural scenarios and we asked the subject to identify what was represented, and/ or to describe what the patient was experiencing or if that image aroused emotions (*emotional evaluation*). At the same time, we boosted cognitive domains such as verbal memory, executive functions, selective and divided attention, using rehabilitative standardized program whom exercise has been chosen ad hoc in relation to the patient's compromised skills and emotional abilities, as described below. Each task was selected by an expert team, specialized in the clinical use of computerized technologies for cognitive rehabilitation.

Moreover, in the other 2 days (ie, on Wednesday and Friday), the patient was submitted to the same CET training, but in a semi-immersive VR scenario by means of BTS-N (VET). BTS-Nirvana is a device with 1 or 2 infrared webcams, 1 workstation touch screen and 1 camera support connected to a big screen or a projector. We chose to use virtual scenarios as Freshness, Water Classic and Storm that represent marine and country landscapes to stimulate the patient's proactivity. In particular, we used “Storm scenario”, characterized by a country landscape with an interactive lawn, aimed at improving visual perceptual ability, besides encouraging the patient to reflect about himself and his daily emotional experience of illness. Each virtual task was built to stimulate the specific emotional domains. The pre and post treatment neuropsychological data are summarized in Table 1.

## 4 Results

The patient did not gain any significant improvement after the conventional approach.

At the end of the combined training, there was a clear improvement in emotional status, with improved ability in identifying and describing his emotions. EOT (the externally oriented cognitive style of thinking) and IMP (the fantasy and IMP) got significantly higher scores. The patient also showed a mild improvement in identifying feelings (difficulty to identifying feelings) and describing feelings/emotions, such as sadness and anger (difficulty to describing feelings): TAS-20= T0:72/100; T1:58/100; TSIA= T0:40/48; T1:28/48. He got also a considerable improvement in mood, with a reduction of depression symptoms and behavioral alterations (Hamilton Rating Scale for Depression = T0:17; T1:12). Furthermore, we observed an increase in cognitive performances, with regard to attention (Montreal Cognitive Assessment Test= T0:25/30; T1: 30/30). Coping strategies also improved, with a significant reduction of voidance strategies, a positive attitude to the emotional management, besides an increase in problem solving skills and social support ability (COPE-NIV = T0:98/228; T1:117/228).

## 5 Discussion

In this case-report, we have observed that the use of innovative technologies can be promising to optimize the cognitive and behavioral recovery in a patient affected by post-stroke alexithymia. In particular, the combined use of CET and VET has proven very effective, in view of the lack of effect obtained from the previous conventional therapy, as shown in Table 1. Alexithymia causes problems in communicating feelings, and therefore it may be considered a factor negatively affecting response to traditional CBT, which instead involves intensive interaction with a clinician and thus empathy. It is possible that this issue is attenuated in computerized CBT, which may make fewer demands in terms of recognizing and communicating affect states and cognitions.^[16]^ Virtual reality, due to the possibility of reproducing ecological environments, provides the patient with the psychological sensation of “being there” boosting the emotional processing. VR has been used for treating different mental disorders such as phobia, eating disorders and schizophrenia, but rarely in patients with neuropsychiatric disorders.^[25]^ Thus, we are not able to state whether VR by itself has played a pivotal role in gaining significant improvement in cognitive and behavioral performance of our patient. To the best of our knowledge, this is the first time ever that innovative rehabilitative tools have been effectively used to manage post-stroke alexithymia. Indeed, to diagnose alexithymia is fundamental to state a prognosis and plan the right therapeutic approach in patients with neurological diseases. This might improve both patients’ quality of life and their caregivers’ psychological well-being.

We are aware that findings from a single case report have many limitations, including epidemiological bias, impossibility of causal inference and generalization and over-interpretation. Thus, our results should be confirmed by well-designed clinical trials, taking into account long-term effects of this promising approach.

Although to date treatment of alexithymia is entrusted to psychotherapy, we believe that advanced trainings, and in particular virtual-mediated protocols, may be effective tools for treating a cluster of post stroke cognitive and emotional sequelae, including alexithymia. However, 1 key issue relevant to the dissemination of advanced cognitive rehabilitation is the identification of those individuals who may benefit from this innovative approach.

## Author contributions

**Conceptualization:** Rocco Salvatore Calabrò, Rosaria De Luca.

**Data curation:** Rosaria De Luca, Alfredo Manuli, Francesca Sciarrone.

**Formal analysis:** Bruno Porcari, Carmela Casella.

**Investigation:** Michele Torrisi, Alfredo Manuli, Francesca Sciarrone.

**Methodology:** Alessia Bramanti, Bruno Porcari, Carmela Casella.

**Project administration:** Rocco Salvatore Calabrò, Rosaria De Luca.

**Resources and Software:** Alessia Bramanti, Bruno Porcari, Carmela Casella, Michele Torrisi.

**Supervision:** Rocco Salvatore Calabrò, Rosaria De Luca.

**Validation:** Rocco Salvatore Calabrò, Rosaria de Luca, Alfredo Manuli, Alessia Bramanti.

**Visualization:** Bruno Porcari, Carmela Casella, Michele Torrisi

**Writing – original draft:** Rocco Salvatore Calabrò, Rosaria De Luca.

**Writing – review & editing:** Rocco Salvatore Calabrò.

## Abbreviations

Abbreviations: CBT = Cognitive behavioral therapy, CET = computerized emotional training, COPE-NIV = coping orientation to problems experienced - New Italian Version, EOT = externally oriented thinking, IMP = imaginative processes, PBA = pseudobulbar affect, TAS = Toronto Alexithymia Scale, TSIA = Toronto structured interview for alexithymia, VET = virtual emotional training.
